# Impact of altitude and anthropogenic disturbance on plant species composition, diversity, and structure at the Wof-Washa highlands of Ethiopia

**DOI:** 10.1016/j.heliyon.2019.e02284

**Published:** 2019-08-12

**Authors:** Fikadu Yirga, Mequannt Marie, Sosina Kassa, Mebrahtu Haile

**Affiliations:** a College of Natural Resource and Environmental Science, Oda Bultum University, Chiro, Ethiopia; b College of Agriculture and Natural Resource, Bonga University, Bonga, Ethiopia; c College of Dryland Agriculture and Natural Resources, Mekelle University, Mekelle, Ethiopia

**Keywords:** Environmental sciences, Ecology, Wof-Washa forest, Disturbance, Plant community, Diversity, Species composition

## Abstract

The study was conducted in Wof-Washa Forest in the central highlands of Ethiopia, aiming at determining the impact of altitude and anthropogenic disturbance on plant species composition, structure, and diversity of the forest. Eighteen transect lines with 632 meters apart from each other were established from top to bottom. A total of 115 main plots for all communities with 20 × 20 m, were established along transect lines from the upper part of the forest to the river's edge. To collect data on seedlings and saplings, 5 m × 5 m and 10 m × 10 m subplots were laid respectively within the main sampling plots. For each plot the plant species were counted, diameter at breast height and height of trees and shrubs were measured. The human disturbance data were visually estimated for each plot in each community. Plant community classification was made following Ethiopia agro-ecological zones. Plant species diversity and richness were found related to human disturbance and altitude. A total of 108 species belonging to 99 genera and 57 families were identified. The results revealed that Asteraceae was the most diverse higher plant family with nine species (8.3%) followed by Fabaceae, Euphorbiaceae, and Rosaceae with six (5.5%) species each. The overall Shannon diversity and evenness index of the forest were 4.02 and 0.86 respectively. Tree/shrub, sapling and seedling densities were 664.4, 757.2 and 805.7 individual's ha^−1^ respectively. The total basal area of the forest was 55.99 m^2^ha^-1^. About 25.7% of the importance values index was contributed by four species, *Juniperus procera, Podocarpus falcatus, Ilex mitis,* and *Erica arborea*. The similarity in species composition within the forest was low, indicating that the different parts of the forest had different floras. The presence of strong human disturbance indicates the need for immediate conservation in order to ensure sustainable utilization and management of the forest.

## 1 Introduction

In recent decades, the areas covered by mountain forests have been two distinct trends, as for forests around the world: continual loss in developing countries (especially in tropical regions) and progressive development in industrialized countries. In Europe, widespread reforestation has happened in numerous mountain regions, related to agricultural land abandonment and declining deforestation, representing around 66% of land cover changes from 1990 to 2006 [1]. However, in some industrialized countries, the expansion of mountain forests has been offset to some extent by losses due to epidemics of diseases and pests or fire [1]. Tropical forests are among the world's ecosystems with the highest species diversity [2]. East African forests are also considered as the center of botanical endemism [3]. Reports by Coetzee [4] and Tamrat [5] revealed that East African mountain forests are among the most diverse and richest African regions with regard to flora composition.

The Ethiopian highlands are considered as one of the most significant countries in Africa with respect to biological resources, both in flora and fauna [6]. They covered large parts of the Afromontane regions of Africa, which stretch from Cameroon to eastern Africa [7], where many biodiversity hotspots exist [8]. Furthermore, the Ethiopian highlands constitute diverse ecological units, extending from moist forest to overall wetlands in the West and Southwest in the direction of Afar depression in the North [9]. The number of species of higher plants such as flowering plants, conifers, and ferns found in the flora of Ethiopia is about 6000, of which about 10% are endemic to the country [10]. As a result, Ethiopia has high levels of biodiversity and it becomes significant for Africa [11].

Although the forests of Ethiopian highlands were characterized by high plant species diversity, they have been reduced and exploited for decades through degradation [12]. This degradation is the outcome of population pressure that increases crop cultivation and livestock grazing in marginal areas. Moreover, agricultural expansion, resettlement systems, charcoal manufacture and persistent extension of actual antagonistic aggressive alive species are taking a deep and determining influence on the plant reserve accessibility [12, 13, 14]. These actions subsidize deforestation and soil erosion on the uplands of the country. Currently, deforestation is estimated to take place at the rate of 160,000–200,000 ha/year [15] which is extremely high. As a result, there has been a rapid decline in the proportion of the forest coverage of the country from 40% in 1900 to 16% in 1954, 8% in 1961, 4% in 1975, 3.2% in 1980, and finally reduced to 2.3% in 2003 [16]. Currently, it is estimated to be 15.7% due to conservation and afforestation campaign launched all over the country in the last ten years [17].

Wof-Washa Forest is among the forests of the Ethiopian highlands, which is registered as one of the National Forest Priority Areas in Ethiopia. The forest is characterized by a high diversity of flora and fauna of the dry Afromontane forests in the country. Although the floristic composition, vegetal community and structural analysis of this forest had been studied so far by Tilahun [18] and Fisaha et al. [19], as in many tropical forests, disturbance (natural and anthropogenic) has been changing the structure and floristic composition of the forest. While Wof-Washa forest is a protected area, it is subjected to human disturbances, resulting in the reduction and a change of the forest cover through time. According to the study conducted by Tilahun [18] and Fisaha et al. [19], deforestation and forest degradation were the major issues in the local area. About 300 hectares of the forest area was completely degraded with very few *Juniperus procera* on the forest border and cliffy bare areas in the higher regions of the forest [18]. This uncontrolled clearing of the forest has been in progress and will continue until efficient management plans are placed to balance the objective of protection, conservation, and sustainable use. Moreover, sufficient data regarding the impacts of altitude and human disturbance on species diversity, composition, and structure were not available in the study area, while they are essential to be documented. Therefore, this calls for the need to generate relevant information in order to make management decisions to protect the forest. Therefore, the objectives of the study are: (I) to evaluate species composition, diversity and structure along an altitude (II) to assess the impact of human disturbance on species composition, diversity, and structure along with the plant communities.

## 2 Materials and methods

### 2.1 Description of the study area

**Fig. 1:**
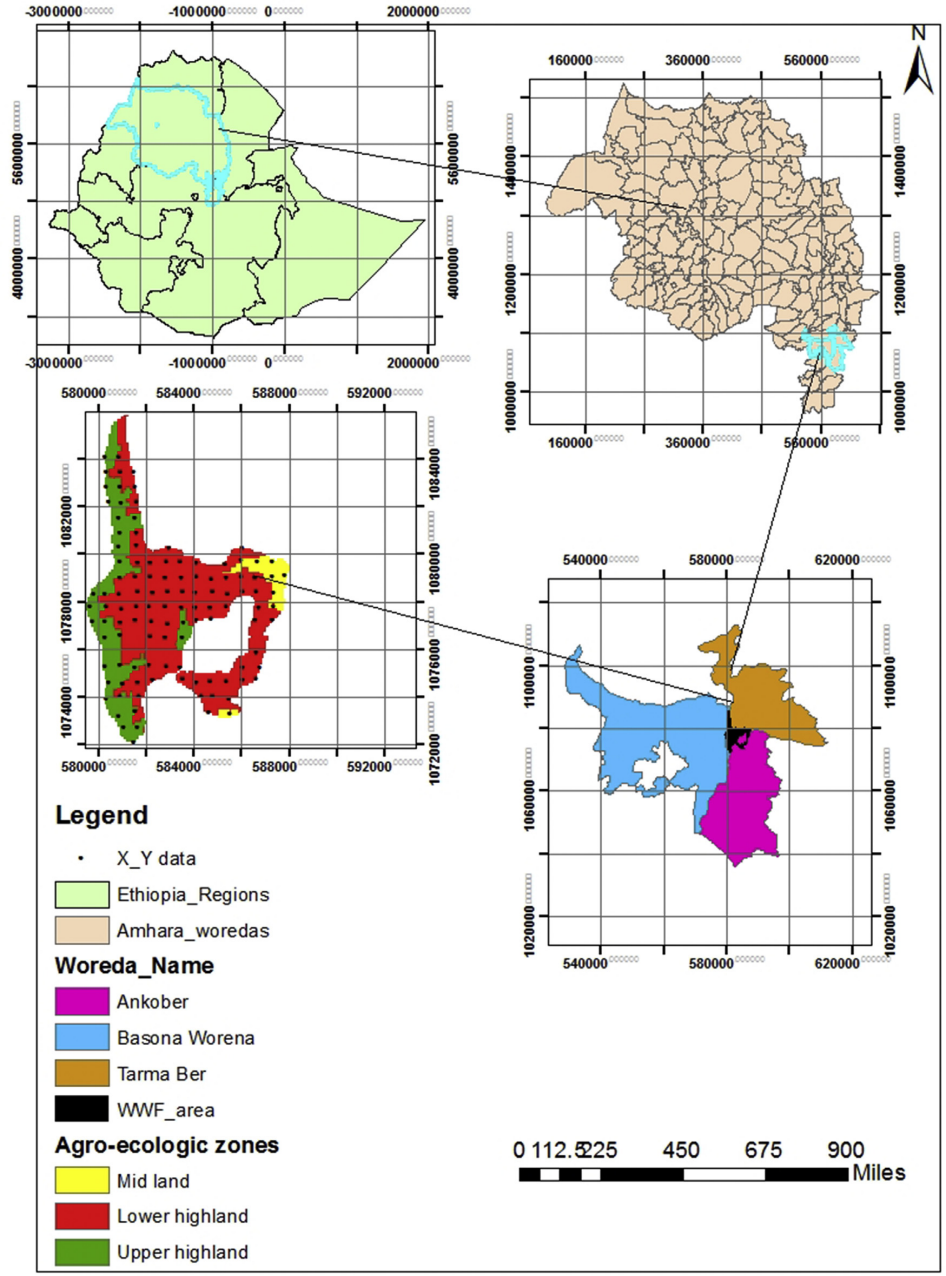
Map of Wof-Washa Forest and distribution of sample plots.

The Wof-Washa forest is located in the Amhara national, regional state, about 60 km far from Debre Berhan town, central highlands of Ethiopia, stretching in three woredas (districts) called Baso, Ankober, and Tarma Ber (Fig. 1). The latitudinal and longitudinal location of the forest is between 9°44′ to 9°46′N and 39°44′ to 39°47′E. The area encompasses an altitude ranging between 1700 m.a.s.l near Gift Michael to 3700 m.a.s.l near Kundi [18]. The forest cover was reduced from 9200 ha since 1994–8200 ha in 2010 and currently, it covers about 7550 ha. The area has the mean annual minimum and maximum temperature that ranges from 11 °C to 20 °C respectively [20]. The rainfall in the area follows a bimodal pattern with a long rainy season between July and September while short rain falls between March and May and the mean annual rainfall is approximately 1400 mm [21].

### 2.2 Methods of data collection

#### 2.2.1 Reconnaissance survey

At the beginning of the study a reconnaissance survey was undertaken and the basic information on the current forest status, site condition, and vegetation distribution were obtained and the possible sampling sites were also determined. During the survey, additional information for the study was also collected from Wof-Washa kebele (the smallest administrative unit) Agricultural Office and from the local communities living close to the study area.

The study area was classified into three plant communities based on Ethiopia agro-ecologic ranges: 1) the midland plant community (1833–2300 m.a.s.l.), 2) the lower highland plant community (2300–3200 m.a.s.l.) and 3) the upper highland plant community (3200–3691 m.a.s.l.) of Wof-Washa forest. The area of each plant community was calculated to take appropriate samples relatively from the three sites based on the area proportion (i.e. larger area takes a high number of samples). Experienced persons were involved during the data collection.

#### 2.2.2 Sampling technique

Systematic sampling technique was applied to gather vegetation data, following Mueller-Dombois & Ellenberg [22]. Eighteen transect lines were laid from the upper portion of the forest area along the river's edge and roads at 632 m apart. The main plots of size 20 m × 20 m were established systematically along these transect lines for trees, shrubs, and lianas. For seedlings and saplings, the sub-plots with 5 m × 5 m and 10 m × 10 m were established respectively, within the main sampling plot.

#### 2.2.3 Vegetation data collection and identification

All plant species encountered in each sample plot were recorded by using their vernacular names. The local names of the species were recorded and included in the list of taxa. The measurement took place for trees and shrubs with the height >2 m and DBH >12.5 cm. The understory of plant species with the height <1.5 m and DBH <2.5 cm were considered as seedlings. Single-stemmed individuals with the height >2 m and DBH >12.5 cm were considered as trees and those in between the seedlings and trees with DBH ≤12. 5 cm and heights of 1.5–2 m were considered as saplings [23].

The diameter at breast height of each tree and shrub was measured 1.3 m above the ground by using tree Caliper and Diameter tape, whereas the height of trees and shrubs were measured by using Merritt-hypsometer and visual estimation. For trees and shrubs that are branched around the breast height, the circumferences were measured separately and then averaged. Trees and shrubs with DBH >12.5 cm were measured and recorded with height and DBH and the conversion of DBH to the basal area was made later. During the study, physiographic variables such as altitude, latitude, and longitude were also measured from the center of each main plot by using the Garmin GPS 60.

Taxonomic identification was made from the flora of Ethiopia and Eritrea [24] and by consulting experts. Voucher specimens were also collected and pressed for identification of the species diversity in the study area and taken to the National Herbarium (ETH), Addis Ababa University, and they were properly identified to species and subspecies levels.

#### 2.2.4 Human disturbance variables

Human disturbance data were visually estimated for each of the main plot (400 m^2^) in each plant community for comparison with the three plant communities of WWF. The type of disturbances was arranged qualitatively [24]. All types of human disturbances were ranked into relatively absent (score 0), low (1), medium (2) and high (3) levels of disturbances (Table 1). The sum of all scores for each plot provides an overall ranking of anthropogenic disturbance in each community. High ranks signify high levels of anthropogenic disturbance and low ranks reveal low levels of disturbance [25].

**Table 1:** Human disturbance variables.

Disturbance	Levels			
	0	1	2	3
Degree of grazing	No Grazing	Low	Medium	High
Fodder	Absent	Low	Medium	High
Medicinal plants	Absent	Low	Medium	High
Timber	Absent	Low	Medium	High
Firewood	Absent	Low	Medium	High

### 2.3 Data analysis

Species diversity, richness, and evenness were determined by using the Shannon-Wiener index [26]. The Shannon-Wiener diversity index, evenness, and richness were determined with respect to the identified species. Principal component analysis (PCA) was performed to show the large pattern over the observed altitudinal gradients and the species overlap between the three plant communities by using an R software package (version 3.6) using vegan packages [27]. The type and degree of human disturbance were analyzed for each community. The scores of each type of disturbance obtained from each plot were summed and averaged. Then the final disturbance levels of each community have been placed to show the highest disturbance rate and absence of disturbance (Table 6).

The quantitative structure of vegetation data was designed based on the analysis of DBH, species density, basal area, height, frequency and Important Value Index (IVI). The DBH and tree height were categorized into DBH and height classes. The relative frequency distribution of individual trees in each plot was calculated. The trees and shrubs relative density and basal area values were calculated on a hectare basis. The importance value indexes (IVI) and basal area (BA) of each tree/shrub species were calculated by using the following equations:

(1) IVI=Relativedensity+Relativefrequency+Relativedominance

Where,

(2) Relativedensity=Numberofindividualspecies/Totalnumberofindividuals×100

(3) Relativefrequency=frequencyofTreespecies/Frequencyofallspecies×100

(4) Relativedominance=Dominanceoftreespecies/Dominanceofallspecies×100

(5) $${Basal\;area=\;\;\pi \;\left(DBH\right)}^{2}$$

Where DBH is diameter at breast height.

The difference in vegetation communities of the forest was determined using analysis of variance (ANOVA) and all vegetation data were tested. One way analysis of variance was used to compare species diversity, evenness, richness, abundance, density, height, DBH and basal area of trees and shrubs of the three plant communities of the forest. The Jaccard's and Sorensen's similarity indices were also used to evaluate the level of species similarities among communities based on their species composition.

## 3 Results

### 3.1 Vegetation composition

One hundred eight plant species belonging to 99 genera and 57 families were recorded in Wof-Washa forest (Table 2). *Asteraceae* was the most species-rich family with nine (8.26%) species; followed by *Fabaceae, Euphorbiaceae,* and *Rosaceae* which contain six species each (5.5% each), whereas *Lamiaceae* had five species (4.6%) and *Poaceae* had four species (3.7%). *Acanthaceae, Rhamnaceae, Rubiaceae,* and *Oleaceae* contributed three species each (2.76% each). Moreover; *Sapindaceae, Rutaceae, Ranunculaceae, Solanaceae, Myrtaceae, Moraceae, Scrophulariaceae, Myrsinaceae, Oleaceae, Anacardiaceae, Polygonaceae, Urticaceae,* and *Cucurbitaceae* had two species each (1.83% each). The remaining 34 families contributed one species each (0.92% each).

**Table 2:** A list of plant species collected from WWF.

Scientific name	Family name	*Local name*	Lifeform
*Acacia abyssinica Hochst. ex Benth.*	*Fabaceae*	*Bazira girar*	T
*Acalypha ornata A. Rich.*	*Euphorbiaceae*	*Nacha*	S
*Acanthus pubescens (Oliv.) Engl*	*Acanthaceae*	*Kosheshila*	S
*Albizia gummifera (J. F. Gmel.) C. A. Sm.*	*Fabaceae*	*Sesa*	T
*Alchemilla pedata A. Rich.*	*Rosaceae*	*Yemdr koso*	H
*Allophylus abyssinicus (Hochst.) Radlk Ofer*	*Sapindaceae*	*Embs*	T
*Aloe vera (L.) Burm.f.*	*Aloaceae*	*Eret*	H
*Artemisia abyssinica Sch.Bip. ex. A. Rich*	*Asteraceae*	*Chikugn*	H
*Arundo donax L.*	*Poaceae*	*Shembeko*	*S*
*Asplenium aethiopicum (Burm.f.) Bech.*	*Aspleniaceae*	*Fern*	F
*Berberis holsti Engl.*	*Berberidaceae*	*Znkila*	S
*Berchemia discolor (Klotzsch) Hemsl.*	*Rhamnaceae*	*Jejeba*	T
*Bersama abyssinica Fresen.*	*Melianthaceae*	*Azamir*	S
*Bridelia micrantha (Hochst.) Baill.*	*Euphorbiaceae*	*Yenebir tifr*	T/S
*Brucea antidysenterica J.F.Mill.*	*Simarubaceae*	*Abalo*	S
*Buddeleja polystachya Fresen.*	*Loganiaceae*	*Anfar*	T
*Calpurnia aurea (Ait.) Benth.*	*Fabaceae*	*Dgta*	S
*Capparis fascicularis*	*Capparidaceae*	*Gumero*	Li/C
*Carissa spinarum L.*	*Apocynaceae*	*Agam*	S
*Casuarina cunninghamiana Miq.*	*Casuarinaceae*	*Arzelibanos*	T
*Celtis africana Burm.*	*Ulmaceae*	*Ameleka*	*T*
*Amaranthus graecizans L.*	*Amaranthaceae*	*Aluma*	H
*Clausena anisata (Willd.) Benth.*	*Rutaceae*	*Lmich*	S
*Clerodendrum myricoides (Hochst) Vatke.*	*Lamiaceae*	*Misrch*	H
*Clematis simensis Fresen*	*Ranunculaceae*	*Azo hareg*	Li/C
*Clutia lanceolata Forssk.*	*Euphorbiaceae*	*Fiyelefej*	S
*Croton macrostachyus Del.*	*Euphorbiaceae*	*Bsana*	T
*Cucumis prophetarum L.*	*Cucurbitaceae*	*embuay*	*H*
*Discopodium penninervium Hochst.*	*Solanaceae*	*Ameraro*	S
*Dodonaea angustifolia L.f.*	*Sapindaceae*	*Kitkita*	S
*Dovyalis abyssinica (A.Rich.) Warb.*	*Flacourtiaceae*	*Koshim*	S
*Echinops kebericho Mesfin.*	*Asteraceae*	*Kebericho*	H
*Ekebergia capensis Sparrm.*	*Meliaceae*	*Lol/sembo*	T
*Eleusine floccifolia (Forssk.) Spreng.*	*Poaceae*	*Akrma*	H
*Embelia schimperi Vatke.*	*Myricaceae*	*Enkoko*	Li/C
*Erythrina brucei Schweinf.*	*Fabaceae*	*Korch/kwara*	T/S
*Erica arborea L.*	*Ericaceae*	*Asta*	S
*Eucalyptus globulus*	*Myrtaceae*	*Nech bahirzaf*	T
*Euphorbia ampliphylla Pax*	*Euphorbiaceae*	*Kulkual*	T
*Euphorbia tirucalli L.*	*Euphorbaceae*	*Knchib*	S
*Ficus sur*	*Moraceae*	*Shola*	T
*Ficus thonningii Blume*	*Moraceae*	*Chibiha*	T/S
*Galiniera saxisfraga (Hochst.) Bridson.*	*Rubiaceae*	*Buna mesay*	S
*Galinsoga quadriradiata Ruiz & Pavon*	*Asteraceae*	*Deha nekay*	*H*
*Galium simense Fresen.*	*Rubiaceae*	*Ashkit*	H
*Guizotia scabra (Vis.) Chiov.*	*Asteraceae*	*Mech*	H
*Hagenia abyssinica (Bruce) J.F. Gmel.*	*Rosaceae*	*Kosso*	T
*Halleria lucida L.*	*Scrophulariaceae*	*Masinkoro*	T/S
*Helicrysum elephantinum Cufod.*	*Asteraceae*	*Nechilo*	S
*Hypericum revolutum Vahl.*	*Hypericaceae*	*Ameja*	S
*Hypoestes forskaolii (Vahl) R.Sch.*	*Acanthaceae*	*Telenj*	H
*Ilex mitis (L.) Radlk.*	*Aquifoliaceae*	*Msar genfo*	T
*Inula confertiflora A.Rich.*	*Asteraceae*	*Weinagift*	S
*Jasminum abyssinicum Hochets. Ex DC.*	*Oleaceae*	*Tenbelel*	Li/C
*Juniperus procera Hochst. ex Endl.*	*Cupressaceae*	*Yehabesha td*	T
*Justicia schimperiana (Hochst. ex Nees) T.*	*Acanthaceae*	*Sensel*	S
*Leonotis raineriana (Burml. f.)*	*Asteraceae*	*Ras kimr*	S
*Lippia adoensis Hochst. ex Walp.*	*Lamiaceae*	*Kessie*	S
*Lobelia rhynchopetalum Hemsl.*	*Lobeliaceae*	*Jibra*	S
*Maesa lanceolata Forssk.*	*Myrsinaceae*	*Kelewa*	T/S
*Maytenus arbutifolia A.*	*Celastraceae*	*Atat*	T/S
*Maytenus obscura (A. Rich.) Cuf.*	*Celasraceae*	*Kumbel*	T/S
*Millettia ferruginea (Hochst.) Bak.*	*Fabaceae*	*Brbira*	T
*Myrica salicifolia A.Rich.*	*Myrtaceae*	*Shinet*	*T/S*
*Myrsine africana L*	*Myrsinaceae*	*Kechemo*	S
*Ocimum lamiifolium Hochst. ex Benth.*	*Lamiaceae*	*Dama kesie*	S
*Olea capensis L. subsp. macrocarpa (C. H. Wright)*	*Oleaceae*	*Damot woira*	T
*Olea europaea .subsp. cuspidata (Wall.ex G. D*	*Oleaceae*	*Woira*	T
*Olinia rochetiana A. Juss.*	*Oliniaceae*	*Tifie*	T/S
*Osyris Quadripartita Dec.*	*Santalaceae*	*Keret*	S
*Otostegia integrifolia A. Rich.*	*Lamiaceae*	*Tinjut*	S
*Peucedanum mattirolii Chiov.*	*Apiaceae*	*Sire Bizu*	*H*
*Phytolacca dodecandra L. Herit.*	*Phytolaccaceae*	*Endod*	Li/C
*Piliostigma thonningii (Schumach.) Milne-Redh.*	*Fabaceae*	*Yekola wanza*	*T*
*Pinus Patula Schiede ex Schltdl.*	*Pinaceae*	*Patula*	T
*Pittosporum viridiflorum Sims.*	*Pittosporaceae*	*Woil*	T/S
*Plantago lanceolata L.*	*Plantaginaceae*	*Gorteb*	H
*Poa leptoclada Hochst. ex A. Rich.*	*Poaceae*	*Dega sar*	H
*Podocarpus falcatus (Thunb.)R.B.ex Mirb.*	*Podocarpaceae*	*Zigba*	T
*Polyscias fulva (Hiern) Harms*	*Araliaceae*	*Yeznjero wober*	T
*Prunus africana (Hook. f.) Kalkm.*	*Rosaceae*	*Tikur enchet*	T
*Psydrax schimperiana (A.Rich.) Bridson*	*Rubiaceae*	*Seged*	T/S
*Ranunculus simensis Fresen.*	*Ranunculaceae*	*Ger hareg*	Li/C
*Rhamnus staddo A. Rich.*	*Rhamnaceae*	*Tsedo*	S
*Rhiocissus Tridentata (L. f.) Wild & Drummond*	*Vitaceae*	*Wodel asfes*	Li/C
*Rhus glutinosa A. Rich.*	*Anacardiaceae*	*Tlem*	T/S
*Rhus vulgaris Meikle*	*Anacardiaceae*	*Yeregna kolo*	T/S
*Ricinus comminus L.*	*Euphorbiaceae*	*Gulo*	S
*Rosa abyssinica Lindley*	*Rosaceae*	*Kega*	S
*Rubus steudneri Schweinf.*	*Rosaceae*	*Enjory*	Li/C
*Rubus volkensii Engl.*	*Rosaceae*	*Yedega enjory*	Li/C
*Rumex abyssinicus Jacq.*	*Polygonaceae*	*Mekmeko*	H
*Rumex nervosus Vahl.*	*Polygonaceae*	*Embuacho*	S
*Salix subserrata Willd.*	*Salicaceae*	*Aheya*	S
*Solanecio gigas (Vatke) C.Jeffrey*	*Asteraceae*	*Shikoko gomen*	H
*Solanum incanum subsp. Adoënse*	*Solanaceae*	*Embuay*	H
*Sparmannia ricinocarpa (J. F. Gmel.) P. B*	*Tiliaceae*	*Wulkifa*	H
*Stephania abyssinica (Dillon & A. Rich.)*	*Menispermaceae*	*Ayt hareg*	Li/C
*Teclea nobilis Del.*	*Rutaceae*	*Atesa/seil*	S
*Thymus schimperi Ronnign.*	*Lamiaceae*	*Tosign*	H
*Urera hypsoledendron (A.Rich.) Wedd.*	*Urticaceae*	*Lankuso*	Li/C
*Urtica Simensis Steudel*	*Urticaceae*	*Sama*	H
*Verbascum sinaiticum Benth.*	*Scrophulariaceae*	*Yahya joro*	H
*Vernonia amygdalina Del.*	*Asteraceae*	*Girawa*	T/S
*Vulpia bromoides (L.) S.F. Gray*	*Poaceae*	*Gofer sar*	H
*Ximenia americana L.*	*Olacaceae*	*Enkoy*	Li/C
*Zehneria scabra (L.F.) Sond.*	*Cucurbitaceae*	*Etse sabek*	*Li/C*
*Ziziphus spina-christi L.*	*Rhamnaceae*	*Kurkura*	*T/S*

T-tree, S-shrub, H-herb, T/S-tree/shrub, Li/C- lianas/climbers and F-fern.

**Fig. 2:**
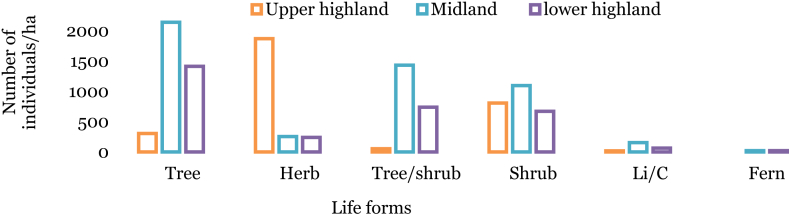
Number of individuals/ha in the plant communities.

Among the total species collected in Wof-Washa forest, tree individuals were found dominant than other plant species with 1164 individuals ha^−1^ followed by shrub (725 ha^−1^), herbs (669 ha^−1^), trees/shrubs (588 ha^−1^), lianas/climbers (63 ha^−1^) and ferns (17 ha^−1^). The midland and lower highland plant communities contain high numbers of tree individuals, whereas, in the upper highland forest community, herbs were found the most dominant species (Fig. 2).

In the midland plant community, the most dominant tree and shrub species were *Casuarina cunninghamiana and Erythrina brucei*, respectively. *Artemisia abyssinica, Jasminum abyssinicum* and *Asplenium aethiopicum* are also the most dominant herb, liana and fern species in this community, respectively (Table 3). Whereas, in the lower highland plant community, *Allophylus abyssinicus* is the most dominant tree followed by *Podocarpus falcatus* and *Juniperus procera. Erica arborea, Vulpia bromoides, Jasminum abyssinicum* and *Asplenium aethiopicum* are the most dominant shrub, herb, liana and fern species in this community respectively (Table 3). However, in the upper highland community of the forest*, Juniperus procera* is the most dominant tree species and *Erica arborea, Poa leptoclada* and *Stephania abyssinica* are the most dominant shrub, herb and liana species respectively (Table 3).

**Table 3:** The abundance of plant species in each plant community.

Species name	Abundance		
	Midland	Lower Highland	Upper Highland
*Casuarina cunninghamiana*	57	255	0
*Erythrina brucei*	55	262	0
*Croton macrostachyus*	54	207	0
*Discopodium penninervium*	54	187	59
*Bersama abyssinica*	44	258	0
*Polyscias fulva*	44	370	0
*Allophylus abyssinicus*	31	389	0
*Podocarpus falcatus*	31	377	0
*Juniperus procera*	37	311	166
*Ilex mitis*	27	309	0
*Poa leptoclada*	0	0	1006
*Thymus schimperi*	0	0	800
*Erica arborea*	0	275	563
*Vulpia bromoides*	0	199	373
*Artemisia abyssinica*	8	71	90
*Asplenium aethiopicum*	14	67	0
*Jasminum abyssinicum*	3	42	0
*Embelia schimperi*	2	32	0
*Amaranthus graecizans*	12	0	0
*Ranunculus simensis*	7	25	0
*Stephania abyssinica*	4	14	3
*Buddeleja polystachya*	0	268	150
*Lobelia rhynchopetalum*	0	0	131

*Acalypha ornate, Berchemia discolor, Capparis fascicularis, Chenopodium ambrosioides,* and another seven plant species were recorded only in the midland community of the forest (Table 3). Whereas, species like *Echinops kebericho*, *Calpurnia aurea, Ekebergia capensis, Halleria lucida* and another six plant species were recorded from the lower highland plant community only (Table 4). On the other hand, plant species such as *Poa leptoclada, Lobelia rhynchopetalum, Helicrysum elephantinum, Thymus schimperi,* and *Rubus volkensii* were recorded only from the upper highland community (Table 4).

**Table 4:** Plant species found solely in each plant community.

Midland plant community	Lower highland plant community	Upper highland plant community
*Chenopodium ambrosioides*	*Calpurnia aurea*	*Poa leptoclada*
*Clutia lanceolata*	*Echinops kebericho*	*Lobelia rhynchopetalum*
*Capparis fascicularis*	*Ekebergia capensis*	*Helicrysum elephantinum*
*Acalypha ornate*	*Halleria lucida*	*Thymus schimperi*
*Berchemia discolor*	*Rumex abyssinica*	*Rubus volkensii*
*Clausena anisata*	*Eucalyptus globulus*	–
*Cucumis prophetarum*	*Pinus patula*	–
*Dodonaea angustifolia*	*Urtica Simensis*	–
*Euphorbia tirucalli*	*Pittosporum viridiflorum*	–
*Ficus sur*	*Berberis holsti*	–
*Olea capensis*	*–*	–

**Fig. 3:**
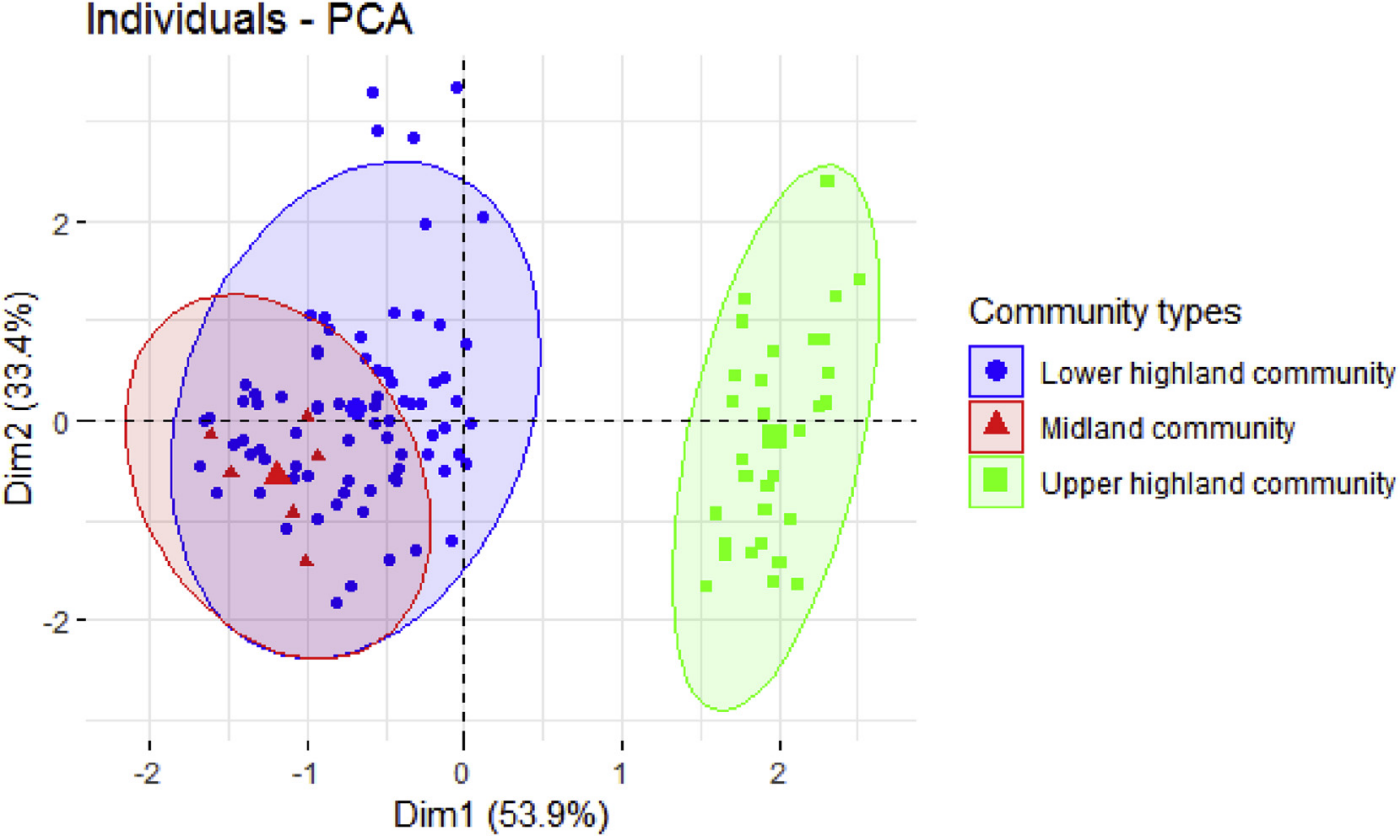
Principal component analysis of species composition across the three plant communities.

Principal component analysis of species composition revealed that the overlap of similar species between the midland and lower highland plant communities. This indicated individuals that are similar are grouped together, and species in the midland community were surrounded by lower highland community plants. However, the surrounding upper highland plant community did not overlap with the adjacent plant community due to the high altitudinal effects (Fig. 3).

### 3.2 Species diversity and richness

The Shannon-Wiener diversity index (H’) was computed for each plant communities and for the overall Wof-Washa forest. Based on the result of the Shannon-Weiner diversity index analysis, the overall plant species diversity and evenness of the forest were found 4.02 and 0.86 respectively. The lower highland plant community of the forest had slightly higher species diversity, evenness, and richness relative to the midland plant community and the upper highland community. Whereas, the upper highland forest community had the highest average altitude interval (3445 m.a.s.l) but had the least species richness, evenness, and diversity (Table 5).

**Table 5:** Species diversity and richness along with the plant communities.

Plant- communities	Altitude (m)	Area (ha)	Area (%)	Number of plots	Species Richness	Diversity index (H′)	H′ max	Species evenness
Midland	1833–2300	242.3	5.4	6	83	3.93	4.41	0.89
Lower highland	2300–3200	3107.3	69.3	78	87	4.01	4.45	0.90
Upper highland	3200–3691	1131.1	25.2	31	37	2.29	3.61	0.63
Overall	1833–3691	4480.7	100	115	108	4.02	4.68	0.86

### 3.3 Human disturbance along with the plant communities

The estimated disturbance levels in the three plant communities varied from a minimum score of 2 for upper highland plant community and a maximum score of 15 for midland plant communities (Table 6). In the midland plant community, all plots were subjected to disturbance whereas, in the lower highland plant community there were 19 control plots with totally undisturbed. Moreover, in the upper highland plant community, 32 control plots were recorded. Arranged in decreasing disturbance scores, the results from the three communities were midland > lower highland > upper highland forest communities. All three communities are subjected to disturbance by cattle and goat browsing and extraction of medicinal plants. Midland community was ranked as highly disturbed in all categories and had a greater disturbance score than lower highland community because of its proximity to human settlements. Even though the human disturbance in the upper highland forest community is very low, the diversity and species richness of this community are also relatively low as it is found in the higher altitudinal gradient.

**Table 6:** Degree of human disturbance along with the plant communities.

Disturbance	Forest communities		
	Midland	Lower highland	Upper highland
Degree of grazing	3	1	1
Fodder	3	2	0
Medicinal plants	3	3	1
Timber	3	1	0
Firewood	3	1	0
Total	15	8	2

### 3.4 Analysis of vegetation structure

#### 3.4.1 Density and frequency distribution of the plant species

The total density of tree/shrub, sapling, and seedling in the WWF were 664.4, 757.2 and 805.7 individuals ha^−1^ respectively. Top five tree species densities in descending order were *Juniperus procera* (52.8 ha^−1^), *Erica arborea* (46.5 ha^−1^), *Allophylus abyssinicus* (32.6 ha^−1^), *Polyscias fulva* (32.2 ha^−1^), *Ilex mitis* (32 ha^−1^) and *Podocarpus falcatus* (30 ha^−1^). The sapling densities in descending order were *Erica arborea* (89.3 ha^−1^), *Juniperus procera* (40.4 ha^−1^), *Polyscias fulva* (31.1 ha^−1^), *Ilex mitis* (31 ha^−1^) and *Buddeleja polystachya* (26.1 ha^−1^) and that of seedling densities were *Erica arborea* (46.3ha-1), *Buddeleja polystachya* (37.4 ha^−1^), *Podocarpus falcatus* (33 ha-1), *Erythrina brucei* (31.5 ha^−1^) and *Olinia rochetiana* (31 ha^−1^).

The frequency of each plant species was revealed that *Juniperus pocera* was the most frequent species (81.7%), followed by *Erica arborea* (61.9%), *Podocarpus falcatus* (58.3%), *Ilex mitis* (55.7%), *Allophylus abyssinicus* (53.9%) and *Buddeleja polystachya* (50.4%). A complete list of species with their frequency and percentage frequency value is presented in Table 7.

**Table 7:** Plants species frequency and relative frequency within each plant community of WWF.

Species name	Lowland		Lower highland		Upper highland		Overall WWF	
	frequency	%	frequency	%	frequency	%	frequency	%
*Acacia abyssinica*	3	1.27	28	1.25	–	–	31	1.09
*Acalypha ornata*	2	0.85	–	–	–	–	2	0.07
*Acanthus pubescens*	–	–	18	0.80	13	3.56	31	1.09
*Albizia gummifera*	3	1.27	39	1.74	–	–	42	1.48
*Alchemida pedata*	1	0.42	17	0.76	7	1.92	25	0.88
*Allophylus abyssinicus*	4	1.69	58	2.58	–	–	62	2.18
*Aloe vera*	1	0.42	14	0.62	–	–	15	0.53
*Artemsia abyssinica*	2	0.85	28	1.25	15	4.11	45	1.58
*Arundo donax*	1	0.42	3	0.13	–	–	4	0.14
*Asplenium aethiopicum*	2	0.85	8	0.36	–	–	10	0.35
*Berberis holsti*	–	–	17	0.76	–	–	17	0.60
*Berchemia discolor*	5	2.12	–	–	–	–	5	0.18
*Bersama abyssinica*	3	1.27	46	2.05	4	1.10	53	1.86
*Bridelia micrantha*	4	1.69	–	–	–	–	4	0.14
*Brucea antidysenterica*	5	2.12	23	1.02	–	–	28	0.98
*Buddeleja polystachya*	–	–	39	1.74	19	5.21	58	2.04
*Calpurnia aurea*	–	–	38	1.69	–	–	38	1.33
*Capparis fascicularis*	2	0.85	–	–	–	–	2	0.07
*Carissa spinarum*	2	0.85	21	0.93	–	–	23	0.81
*Casuarina cunninghamiana*	6	2.54	36	1.60	–	–	42	1.48
*Celtis africana*	3	1.27	26	1.16	–	–	29	1.02
*Chenopodium ambrosioides*	3	1.27	–	–	–	–	3	0.11
*Clausena anisata*	2	0.85	–	–	–	–	2	0.07
*Clematis simensis*	2	0.85	–	–	–	–	2	0.07
*Clerodendrum myricoides*	1	0.42	15	0.67	–	–	16	0.56
*Clutia lanceolata*	2	0.85	–	–	–	–	2	0.07
*Croton macrostachyus*	4	1.69	32	1.42	–	–	36	1.26
*Cucumis prophetarum*	1	0.42	–	–	–	–	1	0.04
*Discopodium penninervium*	4	1.69	33	1.47	14	3.84	51	1.79
*Dodonaea angustifolia*	3	1.27	–	–	–	–	3	0.11
*Dovyalis abyssinica*	3	1.27	20	0.89	–	–	23	0.81
*Echinops kebericho*	–	–	13	0.58	–	–	13	0.46
*Ekebergia capensis*	–	–	18	0.80	–	–	18	0.63
*Eleusine floccifolia*	2	0.85	25	1.11	–	–	27	0.95
*Embelia schimperi*	1	0.42	18	0.80	–	–	19	0.67
*Erica arborea*	–	–	46	2.05	25	6.85	71	2.49
*Erythrina brucei*	4	1.69	49	2.18	–	–	53	1.86
*Eucalyptus globulus*	–	–	8	0.36	–	–	8	0.28
*Euphorbia ampliphylla*	5	2.12	39	1.74	–	–	44	1.55
*Euphorbia tirucalli*	2	0.85	–	–	–	–	2	0.07
*Ficus sur*	3	1.27	–	–	–	–	3	0.11
*Ficus thonningii*	4	1.69	40	1.78	–	–	44	1.55
*Galiniera saxisfraga*	5	2.12	46	2.05	–	–	51	1.79
*Galinsoga quadriradiata*	2	0.85	11	0.49	–	–	13	0.46
*Galium simense*	2	0.85	14	0.62	7	1.92	23	0.81
*Guizotia scabra*	1	0.42	11	0.49	5	1.37	17	0.60
*Hagenia abyssinica*	–	–	32	1.42	16	4.38	48	1.69
*Halleria lucida*	–	–	26	1.16	–	–	26	0.91
*Helicrysum elephantinum*	–	–	–	–	16	4.38	16	0.56
*Hypericum revolutum*	–	–	30	1.34	5	1.37	35	1.23
*Hypoistes forskaolii*	2	0.85	14	0.62	–	–	16	0.56
*Ilex mitis*	4	1.69	60	2.67	–	–	64	2.25
*Inula confertiflora*	–	–	34	1.51	6	1.64	40	1.40
*Jasminum abyssinicum*	2	0.85	19	0.85	–	–	21	0.74
*Juniperus procera*	5	2.12	66	2.94	23	6.30	94	3.30
*Justitia schimperiana*	3	1.27	14	0.62	–	–	17	0.60
*Leonotis raineriana*	2	0.85	–	–	6	1.64	8	0.28
*Lippia adoensis*	3	1.27	29	1.29	8	2.19	40	1.40
*Lobelia rhynchopetalum*	–	–	–	–	16	4.38	16	0.56
*Maesa lanceolata*	4	1.69	49	2.18	–	–	53	1.86
*Maytenus arbutifolia*	2	0.85	39	1.74	–	–	41	1.44
*Maytenus obscura*	3	1.27	31	1.38	–	–	34	1.19
*Millettia ferruginea*	4	1.69	35	1.56	–	–	39	1.37
*Myrica salicifolia*	4	1.69	–	–	11	3.01	15	0.53
*Myrsine africana*	2	0.85	14	0.62	7	1.92	23	0.81
*Ocimum lamiifolium*	–	–	24	1.07	8	2.19	32	1.12
*Olea capensis*	6	2.54	–	–	–	–	6	0.21
*Olea europaea*	4	1.69	41	1.83	–	–	45	1.58
*Olinia rochetiana*	4	1.69	44	1.96	8	2.19	56	1.97
*Osyris quadripartita*	4	1.69	29	1.29	–	–	33	1.16
*Otostegia integrifolia*	2	0.85	10	0.45	–	–	12	0.42
*Peucedanum mattirolii*	3	1.27	10	0.45	6	1.64	19	0.67
*Phytolacca dodecandra*	1	0.42	13	0.58	4	1.10	18	0.63
*Piliostigma thonningii*	3	1.27	–	–	–	–	3	0.11
*Pinus patula*	–	–	30	1.34	–	–	30	1.05
*Pittosporum viridiflorum*	–	–	30	1.34	–	–	30	1.05
*Plantago lanceolata*	1	0.42	12	0.53	3	0.82	16	0.56
*Poa leptoclada*	–	–	–	–	28	7.67	28	0.98
*Podocarpus falcatus*	5	2.12	41	1.83	–	–	46	1.62
*Podocarpus falcatus*	6	2.54	61	2.72	–	–	67	2.35
*Prunus africana*	4	1.69	34	1.51	–	–	38	1.33
*Psydrax schimperiana*	3	1.27	17	0.76	–	–	20	0.70
*Ranunculus simensis*	3	1.27	16	0.71	–	–	19	0.67
*Rhamnus staddo*	2	0.85	18	0.80	–	–	20	0.70
*Rhiocissus Tridentata*	1	0.42	5	0.22	–	–	6	0.21
*Rhus glutinosa*	2	0.85	15	0.67	–	–	17	0.60
*Rhus vulgaris*	4	1.69	41	1.83	–	–	45	1.58
*Ricinus comminus*	2	0.85	15	0.67	5	1.37	22	0.77
*Rosa abyssinica*	2	0.85	19	0.85	7	1.92	28	0.98
*Rubus steudneri*	1	0.42	16	0.71	–	–	17	0.60
*Rubus volkensii*	–	–	–	–	6	1.64	6	0.21
*Rumex abyssinica*	–	–	32	1.42	–	–	32	1.12
*Rumex nervosus*	1	0.42	14	0.62	10	2.74	25	0.88
*Salix subserrata*	4	1.69	40	1.78	–	–	44	1.55
*Solanecio gigas*	2	0.85	10	0.45	1	0.27	13	0.46
*Solanum indicum*	2	0.85	11	0.49	–	–	13	0.46
*Sparmannia ricinocarpa*		0.00	28	1.25	7	1.92	35	1.23
*Stephania abyssinica*	3	1.27	11	0.49	3	0.82	17	0.60
*Teclea nobilis*	2	0.85	28	1.25	–	–	30	1.05
*Thymus schimperi*	–	–	–	–	27	7.40	27	0.95
*Urera pypsoledendron*	–	–	10	0.45	3	0.82	13	0.46
*Urtica Simensis*	–	–	15	0.67	–	–	15	0.53
*Verbascum sinaiticum*	3	1.27	17	0.76	3	0.82	23	0.81
*Vernonia amygdalina*	4	1.69	26	1.16	–	–	30	1.05
*Vulpia bromoides*	–	–	30	1.34	11	3.01	41	1.44
*Ximenia americana*	1	0.42	10	0.45	–	–	11	0.39
*Zehneria scabra*	2	0.85	8	0.36	2	0.55	12	0.42
*Ziziphus spina–christi*	4	1.69	26	1.16	–	–	30	1.05
Total	236	100	2246	100	365	100	2847	100

#### 3.4.2 Diameter at breast height (DBH) distribution of trees and shrub species

**Fig. 4:**
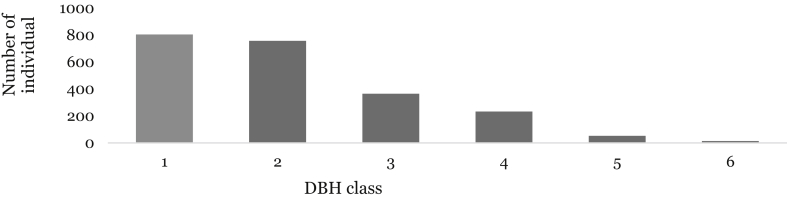
Diameter class distribution of species in WWF. DBH class: (1 = < 2.5 cm; 2 = 2.5–12.5 cm; 3 = 12.6–25 cm; 4 = 25.1–50 cm; 5 = 50.1–80 cm; 6 = >80 cm).

Trees and shrub species were categorized into six DBH classes following Caratti [23]; 1) <2.5 cm, 2) 2.5–12.5 cm, 3) 12.6–25 cm, 4) 25.1–50 cm, 5) 50.1–80 cm and 6) >80 cm. The general pattern of distribution of trees and shrubs in Wof-Washa forest along the different DBH classes seemed to be an inverted J-shaped population distribution. The number of individuals in the forest area decreases significantly from the lowest size classes to the highest size class (Fig. 4).

**Fig. 5:**
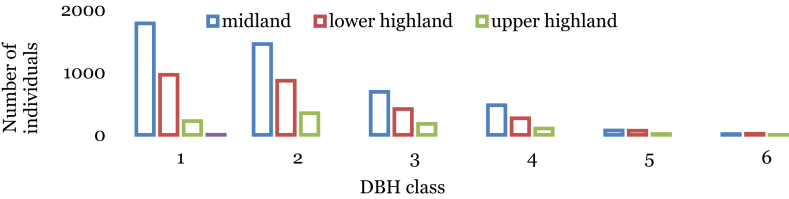
DBH class distribution of species in the plant communities of the forest. DBH class: (1 = < 2.5 cm; 2 = 2.5–12.5 cm; 3 = 12.6–25 cm; 4 = 25.1–50 cm; 5 = 50.1–80 cm; 6 = >80 cm).

Unlike the upper highland plant community, the number of individuals of the midland and lower highland plant communities of the forest areas decreases drastically from the lowest size classes to the highest size class (Fig. 5). However, the majority of individuals of the upper highland plant community were distributed in the second DBH class (Fig. 5). The majority of tree individuals of the midland community were distributed in the first DBH class with 1783 individual's ha^−1^ (39.8%). The distribution of trees in DBH class 2 was 1415 individuals ha^−1^ (32.4%) and 688 (15.3%), 475 (10.6%), 71 (1.6%), 13 (0.3%) individuals ha^−1^ in DBH classes 3, 4, 5 and 6 respectively (Fig. 5). Similarly, the majority of individuals of the lower highland plant community were distributed in the first DBH class with 961 individual's ha^−1^ (37.1%). The distribution of trees in DBH class 2 was 867 individuals ha^−1^ (33.4%) and 414 (16%), 266 (10.3%), 66 (2.5%), 18 (0.7%) individuals ha^−1^ in DBH classes 3, 4, 5 and 6 respectively (Fig. 5). Unlike the two plant communities, the majority of individuals of the upper highland community were distributed in the second DBH class with 347 individuals ha^−1^ (40.3%). The distribution of trees in DBH class 1 was 221 individuals ha^−1^ (25.7%) and 177 (20.5%), 101 (11.7%), 14.5 (1.7%), 2 (0.2%) individuals in DBH classes 3, 4, 5 and 6, respectively (Fig. 5).

#### 3.4.3 Height class distribution of tree and shrub species

**Fig. 6:**
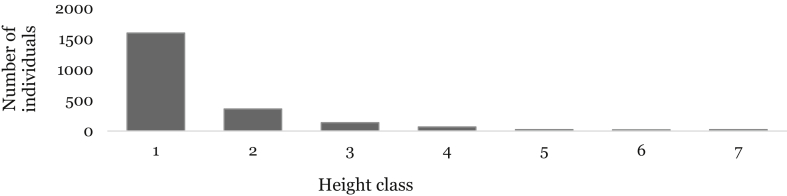
Height class frequency distribution of species in WWF. Height class: (1 = ≤5 m; 2 = 5.1–10 m; 3 = 10.1–15 m; 4 = 15.1–20 m; 5 = 20.1–25 m; 6 = 25.1–30 m; 7 = >30 m).

Tree and shrub individuals recorded in the study area were classified into seven height classes: 1) < 5 m, 2) 5.1–10 m, 3) 10.1–15 m, 4) 15.1–20 m, 5) 20.1–25 m, 6) 25.1–30 m and 7) > 30 m. There were a higher number of tree and shrub individuals in the height class 1, which accounts about 1596.7 individuals ha^−1^ (71.8 %) of the total height classes (Fig. 6).

**Fig. 7:**
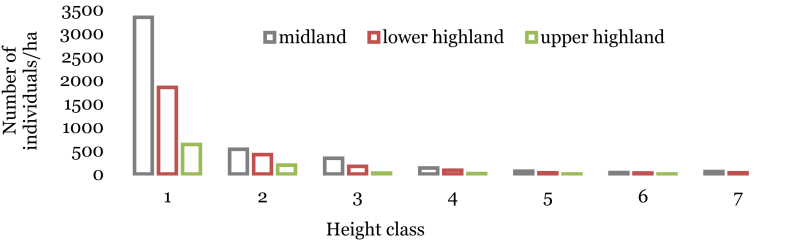
Height class distribution of species in WWF. Height class: (1 = ≤5 m; 2 = 5.1–10 m; 3 = 10.1–15 m; 4 = 15.1–20 m; 5 = 20.1–25 m; 6 = 25.1–30 m; 7 = >30 m).

The highest number of tree individuals in the height class 1 which accounts 3338 individuals ha^−1^ (74.4%) of the total height classes were recorded in the midland forest community (Fig. 7). This appears to be a regular distribution that resembles the inverted J-shaped distribution of individuals in the different height classes with a slight increase in the seventh class (54 individuals ha^−1^), which was higher than the sixth class (33 individuals ha^−1^). Likewise, in the lower highland plant community of the forest, there were very high numbers of tree individuals in the height class 1 which accounts for 1847 individuals ha^−1^ (71.3%) of the total height classes. The upper highland plant community had also a similar distribution of individuals in the two plant communities across the height class, but there was a complete absence of individuals in the seventh height class (Fig. 7).

Tree species that contribute most to the highest height class in the midland community was *Podocarpus falcatus.* Whereas*, Juniperus procera* was the largest tree species, both in the lower highland and upper highland plant communities (Table 8).

**Table 8:** The most abundant tree and shrub species in each class of the three communities.

Height classes	Midland	Lower highland	Upper highland
*Class 1*	*Casuarina cunninghamiana*	*Podocarpus falcatus*	*Erica arborea*
*Class 2*	*Rhus vulgaris*	*Allophylus abyssinicus*	*Erica arborea*
*Class 3*	*Erythrina brucei*	*Ilex mitis*	*Juniperus procera*
*Class 4*	*Prunus africana*	*Polyscias fulva*	*Juniperus procera*
*Class 5*	*Casuarina cunninghamiana*	*Podocarpus falcatus*	*Juniperus procera*
*Class 6*	*Celtis africana*	*Podocarpus falcatus*	*Juniperus procera*
*Class 7*	*Podocarpus falcatus*	*Juniperus procera*	*-*

#### 3.4.4 Basal area (BA) of the plant species and the plant communities

The total basal area of all tree and shrub species was found to be 55.99m^2^ha^-1^. *Juniperus procera* was the dominant species in the forest comprising 16.9% of the total basal area followed by *Podocarpus falcatus* (13%), *Ilex mitis* (7.1%), *Hagenia abyssinica* (6.3%), *Casuarina cunninghamiana* (5.6%), *Euphorbia ampliphylla* (4.9%) and *Polyscias fulva* (4.3%). The basal areas of tree species in the midland, lower highland and upper highland plant communities were found to be 89.2 m^2^ha^-1^, 71.9 m^2^ha^-1^ and 15.9 m^2^ha^-1^ respectively. *Podocarpus falcatus* was the dominant species in the midland community of the forest comprising 18.6% of the total basal area followed by *Prunus africana* (9.9%) and *Polyscias fulva* (7%) (Table 9). On the contrary, *Juniperus procera* was the dominant species in the lower and upper highland communities involving 18.8% and 39.1%, respectively. The second and third dominant species in the lower highland community were *Podocarpus falcatus* (13.1%) and *Ilex mitis* (7.5%) (Table 9).

**Table 9:** BA (m^2^ha^−1^) of top five tree species in each of the plant communities.

Species names	Midland		Lower highland		Upper highland	
	BA	%	BA	%	BA	%
*Podocarpus falcatus*	16.56	18.6	9.43	13.1	–	–
*Prunus africana*	8.81	9.9	–	–	–	–
*Polyscias fulva*	6.26	7.0	–	–	–	–
*Ilex mitis*	6.19	6.9	5.37	7.5	–	–
*Juniperus procera*	5.38	6.0	13.50	18.8	6.21	39.1
*Casuarina cunninghamiana*	–	–	4.34	6.05	–	–
*Euphorbia ampliphylla*	–	–	3.94	5.48	–	–
*Erica arborea*	–	–	–	–	2.11	13.3
*Hagenia abyssinica*	–	–	–	–	4.10	25.9
*Buddeleja polystachya*	–	–	–	–	1.37	8.6
*Discopodium penninervium*	–	–	–	–	0.88	5.6

#### 3.4.5 Important value index (IVI)

According to the IVI of WWF, about 25.7% of the importance values index was contributed by four species, *Juniperus procera, Podocarpus falcatus, Ilex mitis,* and *Erica arborea*. These species were abundant, frequent and dominant in the forest. *Juniperus procera* was found to have the highest IVI (30.7), followed by *Podocarpus falcatus* (21.7), *Ilex mitis (15.9), Erica arborea* (13.1), *Hagenia abyssinica* (12.3) and *Polyscias fulva* (12) (Table 10). The tree species in the forest were grouped into five classes based on their IVI values for conservation priority as follows: class 1) >15, 2) 10.01–15, 3) 5.01–10, 4) 1.01–5 and 5) ≤1 IVI.

**Table 10:** Plant species frequency, relative frequency, density, relative density, basal area and relative dominance of the top ten tree species of the WWF in descending order of IVI.

Species name	BA/ha	RD0m	Frequency	RF (%)	Density/ha	RD (%)	IVI
*Juniperus procera*	9.43	16.85	94	5.93	52.83	7.95	30.7
*Podocarpus falcatus*	7.26	12.97	67	4.23	29.78	4.48	21.7
*Ilex mitis*	3.96	7.08	64	4.04	31.96	4.81	15.9
*Erica arborea*	0.89	1.59	71	4.48	46.52	7.00	13.1
*Hagenia abyssinica*	3.54	6.32	48	3.03	19.35	2.91	12.3
*Polyscias fulva*	2.38	4.25	46	2.90	32.17	4.84	12.0
*Allophylus abyssinicus*	1.19	2.13	62	3.91	32.61	4.91	11.0
*Euphorbiaampliphylla*	2.75	4.92	44	2.78	21.52	3.24	11.0
*Casuarina cunninghamiana*	3.11	5.56	42	2.65	17.17	2.59	10.8
*Buddeleja polystachya*	0.97	1.73	58	3.66	27.39	4.12	9.5

RDom-Relative Dominance; RF-Relative Frequency; RD-Relative Density; IVI- important value index.

In the midland plant community, *Podocarpus falcatus* exhibited the highest IVI (about 27.22), followed by *Prunus africana (*16.43), *Polyscias fulva* (15.3), *Erythrina brucei (*14.04) and *Juniperus procera* (13.3). However, the highest IVI in the lower highland plant community was demonstrated by *Juniperus procera* (29.92) followed by *Podocarpus falcatus* (22.89), *Ilex mitis* (17.78), *Polyscias fulva* (12.94) and *Allophylus abyssinicus* (12.75). In the upper highland community of WWF, *Juniperus procera* exhibited the highest IVI (82.01) followed by *Erica arborea* (69.71), *Hagenia abyssinica* (49.55), *Buddeleja polystachya* (37.62) and *Discopodium penninervium* (24.76).

### 3.5 Species similarity and difference among the plant communities of the forest

The one-way analysis of variance (ANOVA) used in order to check whether there is a significant difference between the three plant communities of the forest along the altitudinal gradient or not were presented as in (Table 11). In post-hoc analysis, Games-Howell's test was also used since equality of variance was not assumed. The post-hoc tests depicted exactly where the differences among the communities have occurred.

**Table 11:** Multiple comparisons between each community in the forest.

Dependent Variable	(I) Community	(J) Community	Mean Difference (I-J)	Std. Error	Sig.
Shannon index	Midland	Lower highland	−.320∗	0.048	0.001
		Upper highland	−1.560∗	0.057	0.000
	Lower highland	Upper highland	−1.240∗	0.038	0.000
Evenness	Midland	Upper highland	−.176∗	0.010	0.000
	Lower highland	Upper highland	−.166∗	0.010	0.000
		Midland	−0.009	0.005	0.182
Richness	Midland	Upper highland	27.425∗	1.759	0.000
	Lower high land	Upper highland	17.040∗	0.503	0.000
		Midland	−27.425∗	1.759	0.000
Abundance/plot	Midland	Upper highland	91.226∗	7.894	0.000
	Lower highland	Upper highland	5.251	5.531	0.612
		Midland	−85.974∗	6.583	0.000
Density/ha	Midland	Upper highland	785.935∗	25.84	0.000
	Lower highland	Upper highland	−57.006∗	4.089	0.000
		Midland	−785.935∗	25.841	0.000
Basal area	Midland	Lower highland	−.025∗	0.007	0.001
		Upper highland	.018∗	0.007	0.034
	Lower highland	Upper highland	.043∗	0.006	0.000
Height of trees	Midland	Lower highland	−0.071	0.459	0.987
		Upper highland	4.493∗	0.467	0.000
	Lower highland	Upper highland	4.564∗	0.259	0.000
Diameter of trees	Midland	Lower highland	−2.380∗	0.921	0.027
		Upper highland	3.284∗	1.062	0.006
	Lower highland	Upper highland	5.664∗	0.752	0.000

∗ The mean difference is significant at the 0.05 level.

The distribution of species among these communities indicated significant dissimilarity; this was observed from the computed Jaccard's and Sorensen's similarity coefficient (Table 12).

**Table 12:** Jaccard's and Sorensen's similarity coefficient within the three communities of the forest.

Plant communities	Jaccard's coefficient			Sorensen's coefficient		
	Midland	Lower highland	Upper highland	Midland	Lower highland	Upper highland
Midland	1			1		
Lower highland	0.65	1		0.44	1	
Upper highland	0.20	0.27	1	0.25	0.31	1

## 4 Discussions

### 4.1 Vegetation composition of the study area

In comparing the vegetation composition of WWF, relatively few species were recorded than other similar Afromontane forests of Kenya such as Kakamega forest (986) [28], Aberdare National Park (778) [29] and Lake Kivu (Rwanda) totally 722 vascular plants [30]; implying that WWF is floristically poorer than these forests (108 vascular plants). But, Kalfou Forest in Cameroon had fewer vascular plants (86) [31] than WWF. The differences in species composition among these forest sites could mainly be attributed to the dissimilarities of the sites in terms of location, altitude, human impact, rainfall, and other biotic and abiotic factors [32].

The vegetation composition of the lower highland plant community (87 species) shows relatively higher species number than midland (83 species) plant community of WWF. However, the vegetation composition of the upper highland plant community (37 species) was founded with very few species than the lower highland and midland plant communities. This variation might be due to the geographic locations of the communities, climatic and edaphic factors and the degree of the human disturbance they have been exposed to [33]. The midland plant community of WWF had also a very low number of plant species than the eastern escarpment of Wollo Ethiopia, situated between 750 and 1780 m.a.s.l in which 216 plant species were analyzed [34].

### 4.2 Species diversity and richness of the plant species

The altitude based classified plant communities were found different in species diversity which indicates the impact of altitude. The higher the species richness resulted in a high evenness which indicates that species richness and evenness were positively correlated. The possible reason for higher diversity and richness of the lower highland community could be it being situated in the inaccessible area for excessive human intervention. In contrast, the midland community of the forest was situated at the lower average altitude interval (2067 m.a.s.l.) which is relatively more favorable for growth and reproduction of a variety of species in the area. However, deforestation and forest degradation are extensively practiced for grazing and agricultural expansion due to the accessibility of the area, and which is closer to the local communities. Moreover, the upper highland plant community had the least species richness, evenness, and diversity that could be associated with growth at a relatively higher altitude in which only better-adapted species potentially grow better than the others. Other studies also revealed that species richness, evenness, and diversity is usually higher in less degraded than degraded sites [35]. The results of the present study are in agreement with the reports from other studies indicated that species richness and diversity tend to be higher at an intermediate altitude and decline at the lower and upper elevations [36].

### 4.3 Human disturbances along with the plant communities

The relationship between the degree of site disturbance and tree species richness is notable among the plant communities. The highly disturbed midland plant community had just 83 species, while the less disturbed site lower highland community had 87 species. These results support the supposition that total species diversity in the dry forest is normally reduced when the disturbance is severe and/or prolonged [37]. Thus the species paucity recorded in the midland forest community could be assigned to the high levels of anthropogenic disturbance (score 15). In several studies, the anthropogenic disturbance has significantly lowered the plant species richness of the dry evergreen forests [38, 39]. It is striking that the 87 species recorded in the moderately disturbed lower highland forest community among the three evergreen forest communities may support the intermediate disturbance hypothesis [40].

### 4.4 Analysis of vegetation structure

#### 4.4.1 Density distribution of the plant species

Comparison of the results of this study with other studies in dry Afromontane forests of the country showed that the density of mature trees/shrubs in WWF is less than Angada forest (4964 individuals ha^−1^) [41], Denkoro forest (811 individuals ha^−1^) [42] and Dodola forest (1293 individuals ha^−1^) [43]. The possible reason for this variation might be due to the presence of high-pressure anthropogenic disturbance as it has been reported by Barnes et al. [44], in which large and medium-size trees have been continuously removed.

#### 4.4.2 Diameter at breast height (DBH) distribution

The general pattern of distribution of trees and shrubs in Wof-Washa forest along the different DBH classes indicates the predominance of small-sized individuals in the forest and similar distribution of tree and shrub species were reported by Fisaha et al. [19], in the same forest. The regular DBH pattern distribution of the midland and the lower highland forest community indicates that the vegetation had good reproduction and low recruitment which might have been due to the selective cutting of large tree individuals as has been stated by Tilahun [18]. However, the irregular DBH distribution (bell-shaped) in the highland community revealed more or less dissimilar vegetation distribution with relative to that of midland and lower highland plant communities.

#### 4.4.3 Basal area of trees and shrubs

The overall basal area of all trees and shrubs in this forest is less than that of Tilahun [18] and Fisaha et al. [19], and, which were 64.32 m^2^ha^-1^ and 360.07 m^2^ha^-1^ respectively. This might be due to the removal of large-sized tree individuals for timber and other construction purposes and the dominance by small-sized trees and shrubs. Other possible reasons for this variation might be due to the difference in the number of sample plots taken and the distance between plots. Moreover, the comparison of the present result of the total basal area of Wof-Washa forest with other related forests shows that it has a lower basal area than Dodola forest (129 m^2^ ha^−1^) [43] and Angoda forest (79.8 m^2^ ha^−1^) [41], but higher than Denkoro forest (45 m^2^ ha^−1^) [42].

Unlike the two other plant communities of the forest, the basal area of trees and shrubs in the upper highland plant community was much less than the two communities and had very few trees and shrubs. For this study, only seven trees and shrub species were recorded in the upper highland community with the DBH and height. This could be due to its harsh environment since environmental variables like altitude, slope, and topography affect the vegetation distribution and excessive erosion was common during the summer season. *Juniperus procera* was the dominant tree species in this community comprising 39.1% of the total basal area followed by *Hagenia abyssinica* (25.9%) and *Erica arborea* (13.3%).

The distribution of plant communities is the manifestation of physical gradients like micro-climate, soil heterogeneity, elevation, biotic response to physical gradients and historical disturbances [11]. As it has been indicated by Tadesse [33], environmental factors such as slope, landscape pattern, and altitude also characterize the distribution of plant communities. Therefore, these environmental factors might influence the plant community formation of the present study in a similar manner.

#### 4.4.4 Important value index (IVI)

The highest basal area of *Juniperus procera* made the species to have a large value of relative dominance and hence got the highest IVI in the forest. In the midland forest community the highest IVI value of *Podocarpus falcatus*, followed by *Prunus africana*, *Polyscias fulva*, *Erythrina brucei* and *Juniperus procera*, indicates that these species were the most dominant and frequent tree species in this community. However, the highest IVI in the lower highland plant community demonstrated by *Juniperus procera*, followed by *Podocarpus falcatus*, *Ilex mitis*, *Polyscias fulva* and *Allophylus abyssinicus* revealed that these species were the most dominant in the lower highland community in the forest.

#### 4.4.5 Species similarity and difference among plant communities of the forest

The Games-Howell's test showed that there was a statistically significant mean difference among the three communities with regard to species diversity, richness and density since the P-value for each community was less than 0.05 alpha level. But in comparing the species evenness, there was no statistically significant mean difference between the midland and lower highland plant community of the forest (P = 0.182). Moreover, in comparing species abundance, there was a statistically insignificant mean difference between the upper highland and the lower highland plant community. This could be due to, the midland and lower highland plant community had similar species evenness yet human disturbance was more practiced in the midland plant community.

There was a statistically significant mean difference between the three communities with regard to basal areas of trees and shrubs (i.e. P = 0.001, less than 0.05). But in comparing the heights of trees and shrubs, there was no statistically significant mean difference between the midland and lower highland community of the forest (i.e. P = 0.987, greater than 0.05). This could be due to species found in both communities may have similar growth and adaptation strategies, but in the midland community, wide-ranging trees were selectively removed illegally relative to the lower highland plant community. Whereas, in comparing the diameters of trees/shrubs, there was a statistical and significant mean difference between the three communities.

Jaccard's (Sj) and Sorensen's (Ss) similarity coefficients were also used to detect vegetation similarities between the three plant communities of the forest. The highest similarity coefficients (Sj = 0.65 and Ss = 0.44) observed between the midland and lower highland plant communities could be due to the fact that the two communities had plots adjacent to each other which indicate similar adaptation mechanisms and requirements of the vegetation. The lowest similarity coefficients (Sj = 0.2 and Ss = 0.25) were observed between the midland and the upper highland plant communities of the forest. The possible reason for this might be mainly due to altitudinal variation and environmental factors in which all plots of the upper highland forest community were located at a higher altitude than plots in the midland community in the forest.

## 5 Conclusions

The analysis of overall vegetation data in Wof-Washa forest indicated the presence of high species diversity, richness, and evenness. From the total species family recorded, *Asteraceae* was the most species-rich family followed by *Fabaceae, Euphorbiaceae,* and *Rosaceae*. The dominance of these families might be due to well-developed strategies and adaptations that would help them to effectively survive in the area. A significant difference regarding all variables in the plant communities along altitudinal gradients was observed. However, the lower highland plant community had the highest species diversity, richness, density, DBH and basal area of trees and shrubs. The variation of these variables could be due to the presence of strong anthropogenic disturbance in the midland plant community for agricultural expansion, selective cutting for charcoal, construction and timber production.

The analysis of vegetation difference among plant communities revealed that altitude had significant effects on species diversity, composition, and structure in Wof-Washa forest. In addition, the human disturbance was found highest in the midland community followed by lower highland and upper highland communities respectively. In the midland and lower highland communities: disturbance, species richness, and diversity were found negatively correlated. The high altitude resulted in a decline of all the variables, especially in the upper highland plant community in the forest. From the structural analysis, the overall diameter and height class distribution patterns of the individuals had a regular (inverted J-shape pattern), reflecting the dominance of small-sized individuals in the lower classes than in the higher classes and resulted in the rare occurrence of large individuals. This is an implication of the existence of excessive cutting of selected size classes in the area. As can be seen from the importance value index of tree species, *Juniperus procera* and *Podocarpus falcatus* were the most dominant tree species in Wof-Washa forest. The present study was delimited to the impacts of altitude and specific human disturbances on species diversity, composition, and structure of plant species and thus, further studies on regeneration status and distribution of plants with respect to other environmental factors like temperature, soil type, and slope are recommended.

## Declarations

### Author contribution statement

Fikadu Yirga, Mequannt Marie, Sosina Kassa, Mebrahtu Haile: Conceived and designed the experiments; Performed the experiments; Analyzed and interpreted the data; Contributed reagents, materials, analysis tools or data; Wrote the paper.

### Funding statement

This research did not receive any specific grant from funding agencies in the public, commercial, or not-for-profit sectors.

### Competing interest statement

The authors declare no conflict of interest.

### Additional information

No additional information is available for this paper.
